# Adaptation of the Safety Climate Survey: A Contribution to Improving Patient Safety

**DOI:** 10.1155/2023/3715960

**Published:** 2023-04-11

**Authors:** Hasan Fehmi Dirik, Seyda Seren Intepeler, Alistair Hewison

**Affiliations:** ^1^Faculty of Nursing, Dokuz Eylul University, Izmir, Turkey; ^2^Institute of Clinical Sciences, University of Birmingham, B15 2TT, Birmingham, UK

## Abstract

**Aims:**

The aim of this study was to adapt the Safety Climate Survey and examine its validity and reliability for use in the Turkish healthcare context.

**Background:**

Maintaining patient safety is a challenge for healthcare systems world-wide, and healthcare professionals need valid and reliable tools to measure improvements in safety.

**Methods:**

The Safety Climate Survey is unidimensional and contains 19 items, which are all five-point Likert-type scales as follows: 1 (totally disagree), 2 (disagree), 3 (neither agree nor disagree), 4 (agree), and 5 (totally agree). Language adaptation of the Safety Climate Survey conducted in accordance with the International Society for Pharma economics and Outcome Research (ISPOR) and expert assessments to calculate the content validity indices was undertaken in the first phase of the study. In phase two, a survey of 434 nurses employed in three hospitals in İzmir (Turkey) was conducted to test the construct validity with confirmatory factor analysis and internal consistency with Cronbach's alpha, split-half reliability, and item-total correlation. The intraclass correlation coefficient was also checked via test-retest reliability for stability.

**Results:**

The content validity index score was 0.97 for the scale and above 0.90 for the items, confirming excellent validity. The confirmatory factor analysis showed an adequate fit, and all the factor loadings were positive and greater than 0.30. Cronbach's alpha was 0.90, and Spearman̶–Brown coefficient 0.83, indicating good internal consistency. The item-total correlation coefficients were between 0.33 and 0.70, exceeding the acceptable level. The intraclass correlation coefficient value obtained was 0.84, reflecting a good level for time stability.

**Conclusion:**

The Turkish version of the Safety Climate Survey is a valid, reliable, and practical tool which can provide essential data on safety issues for healthcare professionals and administrators. *Implications for Nursing Management*. The instrument can be used in hospital settings to measure the safety climate among nurses, and the results obtained can be used to inform the development of safety improvement strategies.

## 1. Introduction

Patient safety concerns the prevention of patient harm caused by healthcare-related errors [1]. However, only 60% of care is based on evidence, or in accordance with guidelines, systems waste about 30% of all health expenditure, and 10% of patients experience harm [2]. The World Health Organization (WHO) also estimated that 1 in 10 patients in high-income countries is harmed while receiving hospital care, and in low and middle-income countries, 134 million adverse events occur, resulting in 2.6 million deaths [3]. This indicates that it is crucial to establish high-quality and safe system designs to provide optimal healthcare services [1].

The prevention of errors in healthcare services and the elimination or reduction of harm require a patient safety culture characterised by continuous improvement involving repetitive evaluations of the safety climate [4, 5]. For a safety culture to be adopted and embedded in institutions, healthcare professionals need to work with the wider health care team in the assessment and improvement of the safety climate [1], and strategies to involve them in decisions and activities that affect them would increase the likelihood of safety initiatives being successful [6]. For example, it would be beneficial for healthcare organizations to formally assess patient safety processes at regular intervals (e.g., every six months) to identify the weaknesses in the system. One element of such an approach could be to examine safety climate [5]. Moreover, developing an approach to organizational learning focused on what works well in complex healthcare systems would result in more rapid improvement than relying solely on conducting investigations when processes fail [7].

The concept of a “safety culture” generally refers to the corporate values, and practices necessary to ensure safety are maintained, whereas the term “safety climate” focuses attention on employee perceptions of how patient safety is defined and managed [5, 8]. Studies which have examined the nature and scope of the safety climate have identified a number of common dimensions. These include the following: leadership commitment to safety, prioritization of safety, teamwork, communication, and safety systems [9–11]. Challenges to the delivery of effective, high-quality, and safe care tend to be the obverse of those listed above including lack of leadership support, staff work pressures, inadequate risk management, communication barriers, and limited resources [12]. Therefore, it is essential to monitor the extent and impact of these factors using robust methods to gather reliable data on safety issues [5]. Tools designed for this purpose include the Safety Attitudes Questionnaire [13], Patient Safety Culture in Healthcare Organizations [14], Hospital Survey on Patient Safety Culture [15], and the Safety Climate Survey [16].

In the present study, the Safety Climate Survey (SCS) was selected because it is unidimensional, yet takes account of complex nature of safety climate, and focuses on institution-wide improvement processes. Its unidimensional format with its 19 items was also a factor in its selection as this was likely to increase the likelihood that participants would complete the survey [5]. A large number of the items with multiple subdimensions in a survey tool scale can be off-putting and result in participants failing to complete an instrument [17]. Hence, the survey length may affect the reliability of the results obtained [18]. The fact that the scale has also been used successfully in different cultures/settings was also a factor in its selection [19].

### 1.1. Aim

The aim of this study was to adapt the SCS and examine its validity and reliability features when applied to the Turkish nursing population.

## 2. Methods

### 2.1. Design

The study was conducted in two phases as given as follows: (1) Translation and language adaptation of the survey. (2) Administration of the survey to nurses working in the Turkish health care settings. The translation and adaptation of instruments for cross-cultural research require rigorous planning and a robust methodological approach. We followed three sets of guidelines [20–22] for the reporting of the psychometric and psycholinguistic properties of the scale.

### 2.2. Setting

To increase the likelihood of the generalizability of the findings, a university hospital and two public hospitals were selected, as providers in other parts of the country were similar in size and structure. The university hospital employed 748 nurses; one public hospital employed 376 nurses, and the other 761 nurses.

### 2.3. Sampling and Participants

It has been recommended that to perform confirmatory factor analysis (CFA) effectively in validity and reliability studies, the number of participants (varying between 100 and 1000) should be at least seven times (in between 3 and 20) the number of items on the scale [23]. For test-retest, the recommended number of participants is between 50 and 100 [24, 25]. Taking these recommendations into account, we aimed to recruit a minimum of 418 participants (20 × 19 items require 380 participants, plus a nonresponse rate of %10 = 418). The final sampling frame was set at 434 nurses for the survey and 82 for the test-retest reliability element.

### 2.4. Measurement/Tool

#### 2.4.1. Safety Climate Survey (SCS)

The unidimensional SCS, developed by Sexton, Helmreich, Pronovost, and Thomas [16], consists of 19 items whose responses are rated on a five-point Likert-type scale. The participants are asked to rate the items of the survey as follows: 1 (totally disagree), 2 (disagree), 3 (neither agree nor disagree), 4 (agree), and 5 (totally agree), 6 (have no idea/comment). The option “I have no idea/comment” is not included in the calculations. Item 18 is reversely scored. The total score is calculated by summing the scores given to all the items and dividing the result by the number of the items. At the end of the procedure, a mean score ranging from one to five is obtained. A score ≥3.75 indicates a positive safety climate perception. The reliability coefficient of the scale was 0.87 in the original study [16]. We also included questions about age, sex, education, length of service, unit worked in, and weekly working hours in addition to the SCS items in order to identify the participants' characteristics.

### 2.5. Data Collection

Nurses were invited to participate if they met the following inclusion criteria: working full-time providing direct patient care for at least one year postqualification. These criteria were necessary because some of the survey items require direct patient care and nursing experience. Nurses not providing direct patient care (e.g., polyclinics) or on sick/annual leave were excluded. A convenience sampling approach was used. During data collection, the researchers visited the hospitals according to the nurses' shifts -initial visit with two additional reminding visits- and distributed the survey to the staff nurses by hand in sealed envelopes. As there was no collection point, the researchers received the completed forms back in sealed envelopes, in person. The returns were given sometimes just immediately after the first visit. In others, it was in a week. Participation was voluntary. The researchers gave information on the survey form and asked participants to fill in it. The final sample who agreed and completed the survey form was 434 in total.

### 2.6. Data Analyses

SPSS 22.0 (IBM Corp., Armonk, NY, USA) and LISREL 8.80 (Lincolnwood, IL: Scientific Software International, Inc.) programs were used for the analysis. Analyses involved confirmatory factor analysis (CFA), split-half method, and item-total correlation, as these have not been undertaken in previous studies. In the assessment of reliability of the instrument for use in the Turkish nursing setting, the test-retest method was used to measure stability, and Cronbach's *α* coefficient, Spearman̶Brown coefficient, and item-total correlation were examined to measure internal consistency. For the validity analyses, content validity was assessed. The CFA was performed for construct validity. The statistical significance level was set at *p* < 0.05 (See Table 1 for a summary of the analytical approaches used).

### 2.7. Translation

In the adaptation process, it is crucial to select idioms and sentence structure that are understandable in the target language but which are consistent with the meaning in the original instrument. This may involve the replacement of particular phrases to ensure they are suitable for the target culture. For this purpose, a pilot study is recommended following the forward and back translations [22].

Forward-back translation was undertaken. Three translators with a good command of English performed the forward translation from the original language (English) to the target language (Turkish). Following forward translation, the researchers (authors), as recommended in the guidelines, checked and modified the items as necessary to eliminate the inconsistencies in the translations (For example, in item 6, “paid attention” was changed to “acted upon”). During the back-translation process from Turkish to English, two different translators, who did not see the scale items beforehand, translated the scales. The researchers then combined the two translations into a single form, and on receipt of approval from its authors [16], a pilot study was conducted with ten nurses to evaluate the items in terms of clarity. In the final part of the translation process, the researchers amended and revised the scale items based on the nurses' recommendations (for example, the wording of item 2, in which “unit” was changed to “clinical area”).

### 2.8. Validity

We used the content validity index for content validity and confirmatory factor analysis for construct validity.

#### 2.8.1. Content Validity

It is recommended that at least 5–8 experts are involved in evaluating the extent to which a survey tool addresses the phenomenon of interest [22, 26] and that the content validity index (CVI) is used [27]. For CVI, the measurement tools are first scored by experts (1 = not relevant, 2 = somewhat relevant, 3 = quite relevant, 4 = highly relevant). Then, the number of experts who gave “three” and “four” points for each item is calculated and then divided by the total number of experts. The result gives the CVI value. The threshold CVI value is 0.78 and above for the scale items (0.75 and above with 10 or more experts) and ideally 0.90 and above for the total scale [28]. In the present study, ten experts (eight nursing faculty members from different departments - experienced in methodological studies, and two clinical nurse managers - one from the ward, one from the critical care unit) assessed the scale items for their suitability. In line with these expert suggestions, the researchers reviewed the scale items' content and made changes in the words used and sentence construction before the survey was conducted [28]. For example, the fifth item was changed after the recommendations, that is, from “There is a leadership approach that focuses on patient safety throughout the institution” to “There is a leadership approach in this institution that directs employees to focus on patient safety.” The CVI was between 0.80 and 1.00 for the scale items and 0.97 for the total scale. We found the CVI value calculated for experts to be 0.97. The expert panel agreed that the scale items are related to the scope of the scale with an agreement rate of over 80% (Table 2). See Table 2 for the CVI indices.

#### 2.8.2. Construct Validity

Construct validity is the level of representation of the factors/items that are related to the construct in the measurement tool [27]. Examining the accuracy of these factors in the scale structure helps determine the suitability of the subdimension items. Two forms of factor analysis (exploratory and confirmatory) and structural equation modeling are the main methods for determining the level of construct validity. While variables in exploratory factor analysis (EFA) produce loadings on all factors, only factors assigned to the model produce loadings in confirmatory factor analysis (CFA). Therefore, CFA is regarded as the approach of choice for the cultural adaptation of measurement tools [29, 30]. Acceptable values for fit indices obtained as a result of CFA are as follows: Chi-square/Degrees of freedom: 2 ≤ 3, Normed Fit Index–NFI: ≥0.90, Comparative Fit Index- CFI: ≥0.95, Incremental Fit Index- IFI: ≥0.95, Goodness of Fit Index–GFI: ≥0.90, and Root Mean Squared Error of Approximation- RMSEA: ≤0.08 [31].

### 2.9. Reliability

We used the test-retest method to measure the stability of the scale. Cronbach's *α* coefficient, split-half analysis, and item-total correlation were selected to determine its internal consistency.

#### 2.9.1. Test-Retest

This involves the measurement of the tool using the same sample group twice at a specified interval [27]. A recommendation for the sample size is between 50 and 100 [24, 25], and the interval between the two measurements should be at least 10–14 days to avoid the possibility of participants remembering the items [32]. The intraclass correlation coefficient (ICC) was used for interpretation with the ICC values being evaluated in four classifications (<0.5: poor, 0.5–0.75: moderate, 0.75–0.9: good, >0.9: excellent) [33].

#### 2.9.2. Cronbach's Alpha

The alpha coefficient (or Cronbach's alpha) is normally calculated to evaluate internal consistency [27]. The range value varies between 0 and 1. High values support the internal consistency of the scale [27]. Ideally, threshold values should be equal to or above 0.70 [34].

#### 2.9.3. Split-Half Method

It is another method for determining levels of internal consistency. It is calculated by dividing the total of items into halves. The Spearman̶Brown coefficient is used [35], and 0.70 and above are regarded as acceptable [34].

#### 2.9.4. Item-Total Correlation

It indicates the items' suitability (whether they will change or not) and the correlation values with the score of each item that is examined. An acceptable level value is above 0.20 [36].

### 2.10. Ethical Considerations

Ethical approval for the conduct of the study was provided by a University Research Ethics Committee (Decision No: 2015/02–20). Agreement to recruit nursing staff was obtained from the executive nursing management of the respective hospitals, and permission via e-mail to use the SCS was confirmed by its developers.

## 3. Results

The mean age of the participants was 35.29 (SD = 7.12), and the majority (92.9%) were female. Two-thirds of the nurses (66.6%) held an undergraduate degree. The distribution of nurses by hospitals was similar in numbers. The average length of professional experience was 13.38 (SD = 7.85) years, and the average weekly working hours were 45.42 (SD = 6.51) (further information about the respondents' socio-demographic details and work-places can be found in Table 3).

### 3.1. Psychometric Properties

#### 3.1.1. Validity

Table 4 shows the confirmatory factor analysis results, presenting the fit indices for the current study findings and acceptable level indices. The scores indicate that the model satisfies the threshold values. Additionally, all the factor loadings of the items were positive and standardized loadings ranged from 0.31 to 0.78, which are above the accepted cut-off value of 0.30.

#### 3.1.2. Reliability

We evaluated the scale's stability with test-retest (two weeks apart). During the analysis, we marked two-way mixed as the model and absolute agreement as the type. We then identified the intraclass correlation coefficient as 0.84 (95% confidence interval, lower limit 0.71, upper limit 0.91) between first and post-test total mean scores (*n* = 82). The results obtained indicate that the time invariance is good.

We tested the internal consistency with Cronbach's alpha, split-half reliability, and item-total correlation. The score for the Cronbach alpha was 0.90 and for Spearman–Brown coefficient 0.83, which are above the acceptable level. Table 4 presents the item-total correlations.

We determined the item-total correlation coefficients to be between 0.33 and 0.70 (acceptable level). The lowest scores are in the first and nineteenth items, and the highest score is in the fifth item (Table 5). The original survey items presented in Table 5 are reproduced from [16].

## 4. Discussion

The analysis demonstrated that the scale has acceptable psycholinguistic and psychometric properties in the Turkish nursing context, which was confirmed on examination of CFA, split-half reliability, and item-total correlations. We discussed the results of the analysis under separate subheadings in the following sections.

### 4.1. Reliability

#### 4.1.1. Stability

As a result of the test-retest performed at two-week intervals, the ICC coefficient in this study was 0.84. Kho et al. [37] determined the ICC value as 0.92 in their study conducted in four intensive care units in a tertiary medical center in Ontario, Canada. The results obtained support the good level of time stability for both studies.

#### 4.1.2. Internal Consistency

In the current study, Cronbach's alpha value was 0.90, and the item-total score correlation coefficients were between 0.33 and 0.70. Kho et al. [37] found the Cronbach's alpha value to be 0.86, and in work with surgical residents in the Netherlands, it was 0.87 [38]. Similarly, a study involving 523 physicians and 1321 nurses working in the operating rooms and surgical services of Swiss hospitals found a value of 0.86 for the German version and 0.84 for the French version [19]. A Cronbach alpha value of 0.70 and above in these adaptation studies in different countries provides evidence for the internal consistency of the scale.

We could not locate an adaptation study comprising split-half reliability and item-total score correlation analysis, and so, these analyses in the present study are the first to provide evidence of these elements of the internal consistency of the scale.

### 4.2. Validity

#### 4.2.1. Content Validity

CVI values were equal to or above 0.80, which exceeded the threshold value of 0.78 in this study. Martowirono et al. [38] focused on the “appropriateness” of the items, in terms of their scale and scope, rather than of the CVI, and resident physicians evaluated the scale items against this criterion. They concluded that the scale items were appropriate for determining the nature of the safety climate. In the same study, the percentage values of the responses of “(6) I have no idea/comment” were calculated for each scale item to support the content validity. The percentage was below 10% for all scale items, and a value of ten percent was considered acceptable [38].

### 4.3. Limitations

Since the data reflect the participants' perceptions, it is important to take this into account when evaluating and interpreting the findings. In addition, nurses working in private hospitals did not participate, and so, caution may be needed when generalising to other settings.

## 5. Implications for Nursing Management

For patient safety to become embedded in the culture of an organisation, all management levels and the healthcare teams need to prioritize patient safety, establish manager-employee cooperation, and regularly revise patient safety policies and procedures and implement them [39]. The present study demonstrates that the SCS is suitable for use in the Turkish healthcare setting and could be used as part of a continuous programme of quality improvement. It is unidimensional, has 19 items, is acceptable to nurses, and helps highlight issues that require action to improve patient safety. It is not a solution but could certainly be part of a comprehensive approach to prioritizing patient safety, leadership, and learning from mistakes [10, 40]. The data also suggest that the SCS can be used successfully in a range of other geographical and health care settings [5, 19, 38].

## 6. Conclusion

The SCS is reliable, has good time stability, a good Cronbach's alpha value, and acceptable item-total correlation coefficients for use in Turkish health care. It is also valid in terms of language, content, and the model fit. These findings reinforce the evidence for the suitability of the scale for use in a range of settings. However, further work is needed to investigate the impact of the use of the SCS on patient safety outcomes.

## Figures and Tables

**Table 1:** Analyses used in the study.

Validity	Reliability
Language adaptation (face validity)	Stability
(i) Forward-back translation	(i) Test-retest
(ii) Pilot testing	
Content validity	Internal consistency
(i) Content validity index	(i) Cronbach's *α*
Construct validity	(ii) Split-half reliability
(i) CFA	(iii) Item-total correlation

**Table 2:** Content validity indices.

Expert–item	E1	E2	E3	E4	E5	E6	E7	E8	E9	E10	Agreement	CVI (agreement/total)
1	+	+	+	+	+	+	+	+	—	+	9	0.90
2	+	+	+	+	+	+	+	+	+	+	10	1.00
3	+	+	+	+	+	+	+	+	+	+	10	1.00
4	+	+	+	—	+	+	+	+	+	+	9	0.90
5	+	+	+	—	+	+	+	+	—	+	8	0.80
6	+	+	+	+	+	+	+	+	+	+	10	1.00
7	+	+	+	+	+	+	+	+	+	+	10	1.00
8	+	+	+	+	+	+	+	+	+	+	10	1.00
9	+	+	+	+	+	+	+	+	+	+	10	1.00
10	+	+	+	+	+	+	+	+	+	+	10	1.00
11	+	+	+	+	+	+	+	+	+	+	10	1.00
12	+	+	+	+	+	+	+	+	+	+	10	1.00
13	+	+	+	+	—	+	+	+	+	+	9	0.90
14	+	+	+	+	+	+	+	+	—	+	9	0.90
15	+	+	+	+	+	+	+	+	+	+	10	1.00
16	+	+	+	+	+	+	+	+	+	+	10	1.00
17	+	+	+	+	+	+	+	+	+	+	10	1.00
18	+	+	+	+	+	+	+	+	+	+	10	1.00
19	+	+	+	+	+	+	+	+	+	+	10	1.00
Agreement	19	19	19	17	18	19	19	19	16	19	CVI item 0.97	
CVI	1.00	1.00	1.00	0.89	0.95	1.00	1.00	1.00	0.84	1.00	CVI expert 0.97	

**Table 3:** Participants' socio-demographic and work-related characteristics (*n* = 434).

Characteristics	*n*	%
Age (35.29 ± 7.12)		
Sex		
Women	403	92.9
Men	31	7.1
Education		
High school graduate	26	6
Associate degree	66	15.2
Bachelor's degree	289	66.6
Graduate degree	53	12.2
Hospital		
University hospital	169	38.9
Public hospital 1	125	28.8
Public hospital 2	140	32.3
Units		
Internal medicine units	171	39.4
Surgical units	174	40.1
Critical care units (intensive care and emergency services)	89	20.5
Tenure (13.38 ± 7.85)		
Weekly working hours (45.42 ± 6.51)		

**Table 4:** Confirmatory factor analysis indices.

Fit indices	Acceptable fit	Present study
*χ*(2)/d.*f*.	2 ≤ 3	3.28
		*χ*(2) = 482.2, d.*f*. = 147, *p* *=* 0.01
NFI	≥0.90	0.95
CFI	≥0.95	0.97
IFI	≥0.95	0.97
GFI	≥0.90	0.90
RMSEA	≤0.08	0.07

**Table 5:** Item-total correlation coefficients.

Items	*r*
(1) The culture of this clinical area makes it easy to learn from the mistakes of others	0.33
(2) Medical errors are handled appropriately in this clinical area	0.63
(3) The senior leaders in my hospital listen to me and care about my concerns	0.52
(4) The physician and nurse leaders in my areas listen to me and care about my concerns	0.67
(5) Leadership is driving us to be a safety-centered institution	0.70
(6) My suggestions about safety would be acted upon if I expressed them to management	0.66
(7) Management/leadership does not knowingly compromise safety concerns for productivity	0.40
(8) I am encouraged by my colleagues to report any safety concerns I may have	0.61
(9) I know the proper channels to direct questions regarding patient safety	0.61
(10) I receive appropriate feedback about my performance	0.52
(11) I would feel safe being treated here as a patient	0.64
(12) Briefing personnel before the start of a shift (i.e., to plan for possible contingencies) is an important part of safety	0.57
(13) Briefings are common here	0.62
(14) I am satisfied with the availability of clinical leadership	0.61
(15) This institution is doing more for patient safety now, than it did one year ago	0.66
(16) I believe that most adverse events occur as a result of multiple system failures, and are not attributable to one individual's actions	0.41
(17) The personnel in this clinical area take responsibility for patient safety	0.66
(18) Personnel frequently disregard rules or guidelines that are established for this clinical area	0.68
(19) Patient safety is constantly reinforced as the priority in this clinical area	0.33

## Data Availability

Data are available from the corresponding author upon request.
